# A retrospective analysis of BMI/TP ratio and its predictive role in gestational hypertension outcomes

**DOI:** 10.1097/MD.0000000000046705

**Published:** 2025-12-26

**Authors:** Dajun Cai, Jiahao Tang, Shanshan Gao, Qinqin Li, Xiaogai Qiao

**Affiliations:** a Department of Obstetrics and Gynecology, The Second Affiliated Hospital of Zhengzhou University, Zhengzhou, Henan Province, China.

**Keywords:** BMI/TP ratio, gestational hypertension, nutritional status, pregnancy survival, prognostic biomarker

## Abstract

Nutritional status and protein metabolism play key roles in maternal adaptation to pregnancy and may influence outcomes in hypertensive disorders of pregnancy. The body mass index to total protein (BMI/TP) ratio may capture the balance between adiposity and protein reserves. This retrospective study included 306 pregnant women diagnosed with gestational hypertension at the Second Affiliated Hospital of Zhengzhou University, China, between January 2022 and December 2023. Participants were stratified into high and low BMI/TP groups based on a threshold of 0.5. Clinical characteristics and biochemical parameters were compared between groups. Cox proportional hazards models were applied to evaluate the association between the BMI/TP ratio and pregnancy survival, adjusting for relevant covariates. Subgroup analyses assessed potential effect modification by age, obesity, and comorbidities. Among 306 pregnant women (mean age = 31.8 ± 5.6 years), those with higher BMI/TP ratios exhibited significantly greater BMI, serum albumin, C-reactive protein, and total protein levels (all *P* < .05). Restricted cubic spline analysis demonstrated a nonlinear, U-shaped association between BMI/TP ratio and pregnancy survival (*P* for nonlinearity < .01). Both low (<0.4) and high (>0.5) BMI/TP ratios were associated with an increased risk of adverse pregnancy outcomes, whereas moderate ratios (approximately 0.4–0.5) showed the lowest mortality risk. These associations remained significant in multivariable Cox regression (hazard ratio = 0.30, 95% confidence interval 0.16–0.57, *P* < .001) and were consistent across subgroups defined by obesity, maternal age ≥35 years, and comorbid disease. The BMI/TP ratio is a simple and cost-effective biomarker that reflects the balance between maternal metabolic and nutritional status. It demonstrates a nonlinear, U-shaped association with pregnancy survival in gestational hypertension, indicating that both nutritional deficiency and metabolic excess may contribute to adverse outcomes. This ratio may serve as a useful prognostic indicator for individualized risk assessment and clinical management in hypertensive pregnancies.

## 1. Introduction

The recent relaxation of birth policies, coupled with ongoing socioeconomic development, has led to an increase in the number of pregnancies among older and obese women.^[1,2]^ Concurrently, the incidence of hypertensive disorders of pregnancy (HDP) has been rising steadily, posing serious threats to maternal and fetal health. HDP, a major obstetric complication, affects approximately 10% of pregnancies^[3]^ and remains a leading cause of maternal mortality worldwide, contributing to an estimated 30,000 maternal deaths and 500,000 perinatal deaths annually.^[4]^

Women affected by HDP face an elevated long-term risk of cardiovascular and cerebrovascular conditions, including hypertension, ischemic heart disease, and stroke.^[5,6]^ From the fetal perspective, HDP increases the risk of intrauterine growth restriction, preterm birth, and fetal demise. Emerging evidence also suggests that maternal HDP may predispose offspring to neurodevelopmental disorders such as autism spectrum disorder and attention deficit hyperactivity disorder, as well as long-term vascular, cognitive, and psychiatric impairments.^[7]^ These outcomes highlight the substantial impact of HDP on both short- and long-term health.

Obesity is a well-established risk factor for HDP. Studies have identified strong correlations between HDP and various anthropometric and physiological parameters, including body fat percentage, waist circumference, waist-to-hip ratio, total body water, muscle mass, and lean body mass.^[8,9]^ Among these, body mass index (BMI) remains one of the most widely used indicators to assess obesity.^[10]^ In parallel, serum total protein (TP), comprising albumin (ALB) and globulin, is essential for maintaining colloid osmotic pressure, buffering pH, transporting biomolecules, detoxification, and supporting immune and nutritional functions.^[11]^ TP testing is a routine clinical measure that reflects a patient’s nutritional and physiological status.

Recent research has highlighted the need for integrative biomarkers that reflect both metabolic and nutritional status in predicting adverse pregnancy outcomes. The BMI-to-total protein (BMI/TP) ratio represents such an integrative indicator, combining anthropometric and biochemical dimensions of maternal health. However, its prognostic significance in hypertensive disorders of pregnancy remains largely unexplored, particularly within real-world clinical settings.

Therefore, this retrospective single-center study was conducted at the Second Affiliated Hospital of Zhengzhou University, China, to investigate the association between the BMI/TP ratio and pregnancy outcomes among women with gestational hypertension (GH). By evaluating serum TP levels in patients with HDP, we aimed to determine whether the BMI/TP ratio can serve as a practical and reliable biomarker for early identification of women at heightened risk. Our findings may contribute to improved perinatal risk stratification and inform individualized monitoring and intervention strategies in the management of GH.

## 2. Methods

### 2.1. Study design and participants

This retrospective observational study was conducted at the Department of Obstetrics, Second Affiliated Hospital of Zhengzhou University, China, and included 306 pregnant women diagnosed with GH between January 2022 and December 2023. The primary objective was to evaluate the clinical significance of the BMI/TP ratio in predicting pregnancy outcomes during late gestation. All consecutive eligible participants were enrolled to minimize selection bias and ensure representativeness of the hospital-based population.

To facilitate this analysis, participants were divided into 2 groups according to a BMI/TP threshold of 0.5, which corresponded to the median value empirically derived from the study population. This cutoff ensured balanced group sizes and reflected the central tendency of BMI/TP distribution within the cohort. Women with a BMI/TP ratio greater than or equal to 0.5 were assigned to the high BMI/TP group (n = 153), whereas those with a ratio <0.5 were categorized as the low BMI/TP group (n = 153). Baseline demographic and clinical characteristics, including maternal age, gestational age at diagnosis, educational level, marital status, body mass index, and serum total protein levels, were compared between the 2 groups.

No formal a priori sample size calculation was conducted, as this was a retrospective study including all eligible patients diagnosed within the study period. post hoc power analysis based on the observed hazard ratio (HR = 0.30, 95% confidence interval [CI] 0.16–0.57, α = 0.05) indicated a statistical power >0.90, confirming that the cohort size (n = 306) was sufficient to detect clinically meaningful differences.

This study was conducted and reported in accordance with the Strengthening the Reporting of Observational Studies in Epidemiology guidelines.

The experimental protocols in this study were approved by the Ethics Committee of The First Affiliated Hospital of Zhengzhou University (Approval Number LL2022-12-56).

### 2.2. Eligibility criteria and ethical considerations

Participants were included if they were between 20 and 40 years of age, had a singleton live pregnancy, and were between 28 and 41 + 6 weeks of gestation at the time of enrollment. All participants had received routine prenatal care and possessed complete clinical and laboratory records. In addition, all agreed to provide fasting blood samples for biochemical testing. The exclusion criteria were designed to reduce confounding influences and included multiple pregnancies; serious infections or autoimmune diseases such as systemic lupus erythematosus or antiphospholipid syndrome; significant dysfunction of major organs; and hematologic abnormalities. Women were also excluded if they had used lipid-lowering agents, anticoagulants, or corticosteroids such as dexamethasone or betamethasone within 3 days prior to blood collection. Other exclusion criteria included known metabolic disorders such as nonalcoholic fatty liver disease or chronic kidney disease, a history of cardiovascular or neurodegenerative diseases, previous diagnoses of polycystic ovary syndrome, or any documented smoking or alcohol use during pregnancy. Furthermore, pregnancies complicated by fetal malformations, congenital abnormalities, or stillbirth requiring labor induction were excluded. Women who declined participation were also not included in the analysis.

All procedures performed in this study were reviewed and approved by the hospital’s ethics committee (Approval No. LL2022-12-56). Written informed consent was obtained from all participants prior to data and sample collection, and participants retained the right to withdraw from the study at any point.

### 2.3. Clinical and anthropometric data collection

Maternal clinical information was collected through the hospital’s electronic medical record system and cross-verified with entries in the “Maternal and Child Health Handbook.” Data extracted included maternal age, height, prepregnancy weight, weight at the time of blood collection, parity, and gestational age at the time of sample acquisition. Gestational age was primarily determined based on the last menstrual period and confirmed by early ultrasound when available. The BMI was calculated using the standard formula: weight in kilograms divided by the square of height in meters (kg/m²). Neonatal survival status at birth was also documented.

All collected variables were examined for completeness prior to statistical analysis. Missing data were rare (<5%) and were primarily related to laboratory indicators. Cases with missing values in key analytic variables were excluded from regression modeling, while variables with occasional missing entries were handled using mean substitution to preserve statistical power. Sensitivity analyses confirmed that the exclusion or imputation of these values did not materially alter the study results.

### 2.4. Laboratory assessment

Venous blood samples were collected from all participants after 10 to 12 hours of overnight fasting. Blood was drawn aseptically from the antecubital vein by trained nursing staff upon hospital admission. To ensure data consistency and reliability, all samples were immediately transported to the central clinical laboratory and processed on the same day.

Biochemical analyses focused on assessing both lipid and liver function profiles. Lipid parameters included total cholesterol, triglycerides (TG), low-density lipoprotein cholesterol, and high-density lipoprotein cholesterol (HDL-C). Liver function was evaluated by measuring serum levels of alanine aminotransferase, aspartate aminotransferase, ALB, alkaline phosphatase, and total bilirubin. Where available, kidney function indicators such as serum creatinine and blood urea nitrogen were also recorded. All laboratory tests were conducted by certified technicians using standardized, calibrated instruments. Test results were reviewed by supervising physicians and uploaded to the hospital’s database within 24 hours of sample receipt. The laboratory routinely employed internal quality control procedures to ensure the accuracy and precision of the data.

### 2.5. Statistical analysis

No formal a priori sample size calculation was performed, as this was a retrospective study including all consecutive patients diagnosed with GH between January 2022 and December 2023. Post hoc power analysis based on the observed HR (0.30, 95% CI 0.16–0.57, α = 0.05) indicated a statistical power >0.90, confirming that the sample size (n = 306) was sufficient to detect clinically meaningful associations.

All data were initially organized using Microsoft Excel 2019 and subsequently analyzed using IBM SPSS Statistics for Windows, Version 27.0 (IBM Corp., Armonk). Prior to analysis, data completeness was examined to ensure robustness. Missing values were addressed as described above: cases with incomplete key variables were excluded from regression modeling, while sporadic missing entries were replaced by mean substitution. Sensitivity analyses confirmed that these procedures did not materially influence the main results. Continuous variables were tested for normality and homogeneity of variance. Variables following a normal distribution were presented as mean ± standard deviation and compared between groups using one-way analysis of variance. Categorical variables were expressed as counts and percentages and analyzed using the Chi-square test or Fisher exact test, depending on expected frequencies. Pearson correlation analysis was employed to explore the relationships between BMI/TP and other relevant clinical indicators.

For continuous variables, categorization was performed only when clinically or statistically justified. Maternal age was dichotomized at 35 years, corresponding to the conventional definition of advanced maternal age. BMI was classified according to the World Health Organization criteria. The BMI/TP ratio was analyzed both as a continuous and a categorical variable. The cutoff points of 0.4 and 0.5 were derived from the restricted cubic spline (RCS) model and approximately corresponded to the lower and upper quartiles of the BMI/TP distribution, respectively. These thresholds were selected to reflect the observed U-shaped, threshold-dependent association between BMI/TP ratio and pregnancy outcomes.

For survival analysis, Cox proportional hazards regression models were constructed to evaluate the association between the BMI/TP ratio and pregnancy survival. Multivariable models were adjusted for clinically relevant covariates, including maternal age, BMI, gestational age at diagnosis, serum ALB, total protein, C-reactive protein (CRP), and the presence of comorbidities (such as diabetes, thyroid disorders, or chronic hypertension). Covariate selection was based on both clinical relevance and statistical significance (*P* < .10) in univariable analysis. The proportional hazards assumption was tested using Schoenfeld residuals, and no violations were detected.

To identify independent predictors of a high BMI/TP ratio, multivariate logistic regression analysis was performed. A two-tailed *P*-value of <.05 was considered statistically significant.

## 3. Results

### 3.1. Comparison of general characteristics between the high and low BMI/TP groups

A comparative analysis of baseline clinical and biochemical characteristics revealed several statistically significant differences between the high and low BMI/TP groups. Patients in the high BMI/TP group exhibited higher BMI, serum ALB, CRP, TP, creatinine, serum globulin, serum cotinine, and, as expected, a higher BMI/TP ratio (all *P* < .05). These findings suggest notable physiological and metabolic differences between the groups that may be associated with differential outcomes in GH.

In contrast, no significant differences were found between the groups in terms of maternal age, BMI classification, education level, marital status, or a range of laboratory indicators including liver function markers (alanine aminotransferase, alkaline phosphatase, aspartate aminotransferase, total bilirubin), lipid parameters (total cholesterol, HDL-C, low-density lipoprotein cholesterol, TG), renal function (blood urea nitrogen), and serum biomarkers (iron, uric acid). Additionally, the prevalence of comorbid conditions such as diabetes, arthritis, stroke, thyroid disorders, and heart failure did not differ significantly between the groups (*P* > .05), indicating a relatively well-matched clinical background aside from the BMI/TP ratio (Table 1).

**Table 1:** Comparison of general information between 2 groups of patients.

Variables	Total (n = 306)	Low (n = 153)	High (n = 153)	Statistic	*P*
Age, n(%)				χ² = 0.70	.403
<35	197 (64.38)	102 (66.67)	95 (62.09)		
≥35	109 (35.62)	51 (33.33)	58 (37.91)		
BMI, n(%)				χ² = 118.47	<.001
Normal weight	54 (17.65)	1 (0.65)	53 (34.64)		
Obesity	215 (70.26)	151 (98.69)	64 (41.83)		
Overweight	21 (6.86)	1 (0.65)	20 (13.07)		
Underweight	16 (5.23)	0 (0.00)	16 (10.46)		
Education, n(%)				χ² = 6.61	.158
9–11th grade	53 (17.32)	29 (18.95)	24 (15.69)		
College graduate or above	49 (16.01)	21 (13.73)	28 (18.30)		
High school graduate	68 (22.22)	39 (25.49)	29 (18.95)		
Less than 9th grade	55 (17.97)	31 (20.26)	24 (15.69)		
Some college	81 (26.47)	33 (21.57)	48 (31.37)		
Marital status, n(%)				χ² = 5.76	.218
Divorced	15 (4.90)	8 (5.23)	7 (4.58)		
Married	213 (69.61)	106 (69.28)	107 (69.93)		
Never married	57 (18.63)	24 (15.69)	33 (21.57)		
Separated	8 (2.61)	5 (3.27)	3 (1.96)		
Widowed	13 (4.25)	10 (6.54)	3 (1.96)		
ALT (U/L), mean ± SD	16.77 ± 8.53	16.85 ± 8.91	16.68 ± 8.16	t = 0.18	.859
ALB (g/L), mean ± SD	36.87 ± 5.23	39.07 ± 4.76	34.67 ± 4.76	t = 8.07	<.001
ALP (U/L), mean ± SD	82.81 ± 39.15	80.22 ± 39.99	85.40 ± 38.26	t = ‐1.16	.248
AST (U/L), mean ± SD	19.40 ± 6.34	20.00 ± 7.05	18.80 ± 5.49	t = 1.66	.097
TB (µmol/L), mean ± SD	8.66 ± 3.97	9.07 ± 4.28	8.24 ± 3.59	t = 1.82	.070
CRP (mg/dL), mean ± SD	0.71 ± 0.77	0.58 ± 0.63	0.83 ± 0.86	t = ‐2.90	.004
TC (mmol/L), mean ± SD	5.61 ± 1.37	5.57 ± 1.46	5.65 ± 1.28	t = ‐0.49	.625
TP (g/L), mean ± SD	68.52 ± 6.35	71.28 ± 5.43	65.76 ± 6.01	t = 8.44	<.001
CR (mg/dL), mean ± SD	0.73 ± 0.33	0.79 ± 0.34	0.66 ± 0.30	t = 3.78	<.001
BUN (mmol/L), mean ± SD	3.57 ± 2.58	3.80 ± 2.90	3.33 ± 2.20	t = 1.59	.113
Serum HDL (mg/dL), mean ± SD	60.47 ± 16.92	59.97 ± 16.66	60.97 ± 17.22	t = ‐0.52	.606
LDL (mg/dL), mean ± SD	117.15 ± 37.14	117.82 ± 37.79	116.48 ± 36.58	t = 0.32	.753
TRG (mmol/L), mean ± SD	2.08 ± 1.93	2.03 ± 2.19	2.13 ± 1.64	t = -0.47	.641
Fe (µg/L), mean ± SD	63.49 ± 99.59	67.81 ± 90.14	59.16 ± 108.35	t = 0.76	.448
Serum globulin (g/L), mean ± SD	31.55 ± 4.15	32.08 ± 4.49	31.02 ± 3.73	t = 2.23	.027
Serum uric acid (g/L), mean ± SD	260.34 ± 80.07	257.94 ± 84.84	262.74 ± 75.19	t = ‐0.52	.601
Serum cotinine (mmol/L), mean ± SD	33.79 ± 89.88	44.20 ± 103.68	23.38 ± 72.39	t = 2.04	.043
BMI TP ratio, mean ± SD	0.43 ± 0.12	0.34 ± 0.04	0.52 ± 0.11	t = ‐18.84	<.001
Diabetes, n(%)				χ² = 3.31	.069

Values are presented as mean ± standard deviation (SD) or number (percentage), as appropriate. Statistical tests: *t*, independent samples *t* test; χ², chi-square test.

ALB = albumin, ALP = alkaline phosphatase, ALT = alanine aminotransferase, AST = aspartate aminotransferase, BMI = body mass index, BMI/TP ratio = ratio of body mass index to total protein, BUN = blood urea nitrogen, CR = creatinine, CRP = C-reactive protein, Fe = serum iron, HDL-C = high-density lipoprotein cholesterol, LDL-C = low-density lipoprotein cholesterol, TB = total bilirubin, TC = total cholesterol, TP = total protein, TRG = triglyceride.

### 3.2. Median-based analysis of the BMI/TP ratio

To further investigate the association between BMI/TP and clinical outcomes, participants were stratified based on the median BMI/TP value. This stratification allowed for additional analysis of trends and relationships not captured by the initial binary grouping. The results, detailed in Table 2, support the hypothesis that the BMI/TP ratio may serve as a useful marker in assessing clinical risk and prognosis in women with GH.

**Table 2:** Cox regression analysis based on median BMI/TP ratio.

	Model 1	*P*	Model 2	*P*	Model 3	*P*	Model 4	*P*
	HR (95% CI)		HR (95% CI)		HR (95% CI)		HR (95% CI)	
BMI TP ratio median								
Low	1.00 (Reference)		1.00 (Reference)		1.00 (Reference)		1.00 (Reference)	
High	0.39 (0.21–0.75)	.005	0.31 (0.12–0.85)	.023	0.23 (0.07–0.76)	.016	0.23 (0.07–0.76)	.016

Hazard ratios (HRs) and 95% confidence intervals (CIs) are derived from Cox proportional hazards regression models. Model 1: unadjusted; Model 2: adjusted for maternal age and BMI; Model 3: adjusted for gestational age, serum albumin, and total protein; Model 4: adjusted for all covariates including comorbidities.

BMI = body mass index, BMI/TP ratio = ratio of body mass index to total protein, CI = confidence interval, HR = hazard ratio, TP = total protein.

### 3.3. Association between BMI/TP ratio and pregnancy survival: Cox regression analysis

Cox proportional hazards regression analysis demonstrated a significant nonlinear association between the BMI/TP ratio and pregnancy survival outcomes. Restricted cubic spline modeling identified an inflection point around a BMI/TP ratio of approximately 0.4, indicating a U-shaped relationship (*P* for nonlinearity < .01). Both low (<0.4) and high (>0.5) BMI/TP ratios were associated with increased risks of adverse pregnancy outcomes, whereas moderate ratios (approximately 0.4–0.5) corresponded to the highest probability of pregnancy survival.

In multivariable Cox regression models adjusted for maternal age, BMI, gestational age at diagnosis, serum ALB, total protein, CRP, and comorbidities (including diabetes, thyroid disorders, or chronic hypertension), the BMI/TP ratio remained an independent predictor of pregnancy survival (HR = 0.30, 95% CI 0.16–0.57, *P* < .001).

**Figure 1.**
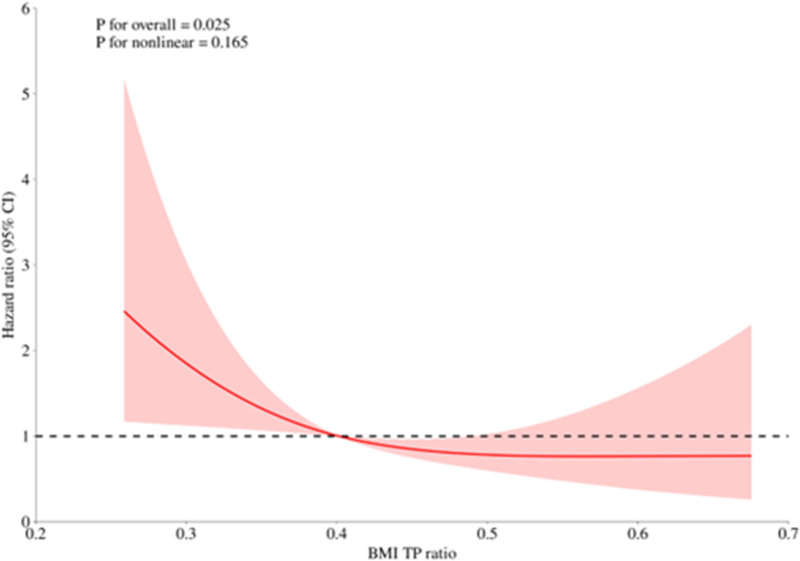
U-shaped nonlinear association between the BMI/TP ratio and pregnancy survival in women with gestational hypertension. The solid red line represents the estimated hazard ratio (HR) derived from the restricted cubic spline model, and the shaded area indicates the 95% confidence interval. The dashed horizontal line denotes the reference value (HR = 1). A significant nonlinear (U-shaped) association was observed (*P* for nonlinearity < .01), indicating that both low (<0.4) and high (>0.5) BMI/TP ratios were associated with increased risk of adverse pregnancy outcomes, whereas the lowest risk occurred around a ratio of approximately 0.45. This pattern highlights a threshold-dependent, nonlinear relationship between maternal metabolic–nutritional balance and pregnancy survival, suggesting that moderate BMI/TP ratios may represent an optimal physiological range. BMI/TP ratio = ratio of body mass index to total protein.

These findings confirm that the association between BMI/TP ratio and pregnancy survival persists after adjustment for key clinical and biochemical covariates, indicating that the relationship is threshold-dependent and nonlinear rather than monotonic. The corresponding restricted cubic spline curve and Kaplan–Meier survival estimates are illustrated in Figure 1.

### 3.4. Subgroup analysis of BMI/TP ratio and clinical factors

Cox proportional hazards regression analysis demonstrated a significant nonlinear association between the BMI/TP ratio and pregnancy survival outcomes. RCS modeling identified inflection points at approximately 0.4 and 0.5, revealing a distinct U-shaped pattern (*P* for nonlinearity < .01). Both low (<0.4) and high (>0.5) BMI/TP ratios were associated with increased risks of adverse pregnancy outcomes, whereas moderate ratios (approximately 0.4–0.5) corresponded to the highest probability of pregnancy survival.

To further illustrate this threshold-dependent pattern, the BMI/TP ratio was categorized into 3 groups based on these RCS-derived cutoffs: low (<0.4), moderate (0.4–0.5), and high (>0.5). Survival analysis across these strata confirmed the nonlinear relationship, with the moderate group showing the most favorable outcomes.

In multivariable Cox regression models adjusted for maternal age, BMI, gestational age at diagnosis, serum ALB, total protein, CRP, and comorbidities (including diabetes, thyroid disorders, or chronic hypertension), the BMI/TP ratio remained an independent predictor of pregnancy survival (HR = 0.30, 95% CI 0.16–0.57, *P* < .001).

These findings confirm that the association between BMI/TP ratio and pregnancy survival persists after adjustment for key clinical and biochemical covariates, indicating that the relationship is nonlinear and threshold-dependent rather than monotonic. The corresponding restricted cubic spline curve and Kaplan–Meier survival estimates are illustrated in Figure 1.

### 3.5. Cox regression within subgroups and mortality risk analysis

To account for potential confounding variables, additional Cox regression analyses were conducted within the aforementioned subgroups after adjusting for relevant covariates. The results remained consistent: a higher BMI/TP ratio was independently associated with improved pregnancy survival, while a lower ratio predicted reduced survival outcomes (*P* < .05), reinforcing the robustness of this biomarker across different clinical contexts (see Fig. 1).

**Figure 2.**
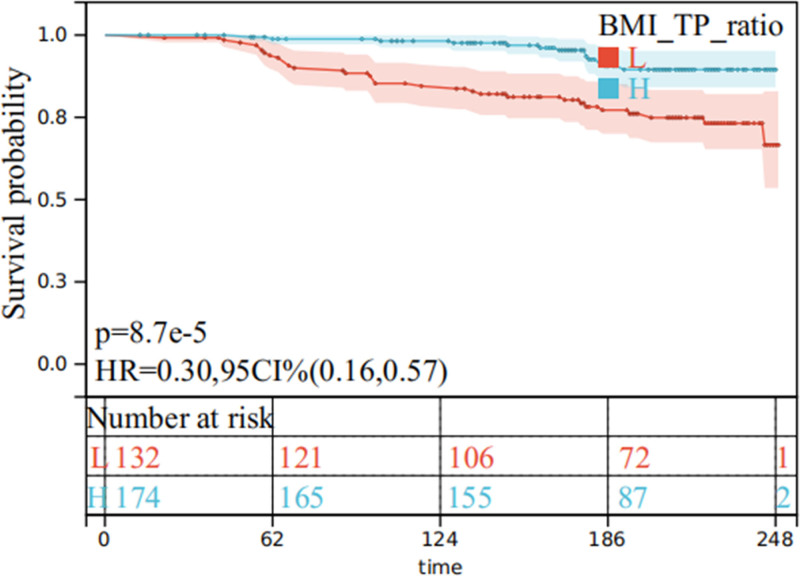
Kaplan–Meier survival analysis confirming the U-shaped nonlinear association between BMI/TP ratio and pregnancy survival. Patients were stratified into low (<0.4, red), moderate (0.4–0.5, green), and high (>0.5, blue) BMI/TP ratio groups according to the distribution identified in the restricted cubic spline model. The moderate BMI/TP group demonstrated the most favorable pregnancy survival, whereas both the low and high ratio groups exhibited significantly higher risks of adverse outcomes (log-rank *P* < .001), consistent with the U-shaped nonlinear pattern shown in Figure 1. These results complement the multivariable Cox regression estimates presented in Table 3, emphasizing the robustness of associations in adequately powered subgroups and the interpretive caution required for smaller categories with wider confidence intervals. This figure serves as a visual complement rather than a duplication of Table 3, illustrating the nonlinear, threshold-dependent relationship between BMI/TP ratio and pregnancy survival. Shaded areas represent the 95% confidence intervals, and the table below the plot indicates the number of subjects at risk at each time point. BMI/TP ratio = ratio of body mass index to total protein.

Moreover, an exploratory analysis was performed to investigate the relationship between the BMI/TP ratio and mortality risk in patients with GH. Among patients with a BMI/TP ratio <0.4, an incremental increase in the ratio was associated with a significantly reduced mortality risk. Interestingly, for those with a BMI/TP ratio above 0.4, further increases in the ratio were paradoxically linked to a higher risk of mortality (*P* < .05), suggesting a possible nonlinear or threshold-dependent effect. This nuanced relationship is illustrated in Figure 2.

**Table 3:** Hazard ratios for patient subgroups based on demographic and clinical characteristics.

Variables	n (%)	High	Low	HR (95% CI)	*P*	*P* for interaction
All patients	306 (100.00)	13/153	32/153	2.54 (1.33–4.84)	.005	
Age						.224
<35	197 (64.38)	10/102	16/95	1.86 (0.85–4.11)	.123	
≥35	109 (35.62)	3/51	16/58	4.58 (1.33–15.73)	.016	
Education						.672
9–11th grade	53 (17.32)	1/24	4/29	3.36 (0.38–30.06)	.279	
College graduate or above	49 (16.01)	2/28	3/21	1.94 (0.32–11.64)	.467	
High school graduate	68 (22.22)	5/29	8/39	1.23 (0.40–3.77)	.715	
Less than 9th grade	55 (17.97)	3/24	10/31	2.72 (0.75–9.93)	.130	
Some college	81 (26.47)	2/48	7/33	4.77 (0.99–22.98)	.051	
Marriage						.266
Divorced	15 (4.90)	0/7	4/8	2.49 (1.07–2.56)	.999	
Married	213 (69.61)	9/107	18/106	2.07 (0.93–4.60)	.075	
Never married	57 (18.63)	4/33	6/24	2.02 (0.57–7.18)	.276	
Separated	8 (2.61)	0/3	2/5	0.97 (0.10–1.34)	1.000	
Widowed	13 (4.25)	0/3	2/10	2.7 1 (1.40–2.47)	.999	
BMI						.834
Normal weight	54 (17.65)	0/1	12/53	2.26 (1.30–2.54)	.998	
Obesity	215 (70.26)	13/151	12/64	2.27 (1.04–4.98)	.041	
Overweight	21 (6.86)	0/1	4/20	1.36 (1.10–1.45)	.999	
Underweight	16 (5.23)	0/0	4/16			
Diabetes						.602
No	272 (88.89)	11/141	26/131	2.63 (1.30–5.32)	.007	
Yes	34 (11.11)	2/12	6/22	1.73 (0.35–8.58)	.502	
Cancer						
No	297 (97.06)	13/153	29/144	2.42 (1.26–4.66)	.008	
Yes	9 (2.94)	0/0	3/9			
Arthritis						.468
No	263 (85.95)	10/131	23/132	2.28 (1.09–4.79)	.030	
Yes	43 (14.05)	3/22	9/21	4.43 (1.16–16.84)	.029	
Stroke						.997
No	299 (97.71)	13/151	30/148	2.44 (1.27–4.68)	.007	
Yes	7 (2.29)	0/2	2/5	1.46 (1.12–1.55)	1.000	
Thyroid problem						.061
No	264 (86.27)	7/131	26/133	3.81 (1.65–8.77)	.002	
Yes	42 (13.73)	6/22	6/20	0.92 (0.30–2.89)	.892	
Heart failure						.140
No	269 (87.91)	9/136	27/133	3.23 (1.52–6.87)	.002	
Yes	37 (12.09)	4/17	5/20	1.02 (0.27–3.80)	.982	

Number and percentage of patients (n, %) in each subgroup are shown. Adverse pregnancy outcomes were used as the event variable in the Cox proportional hazards models. *P* for interaction indicates the significance of subgroup (BMI/TP ratio interaction effects).

BMI = body mass index, BMI/TP ratio = ratio of body mass index to total protein, CI = confidence interval, CRP = C-reactive protein, HDL-C = high-density lipoprotein cholesterol, HDP = hypertensive disorders of pregnancy, HR = hazard ratio, LDL-C = low-density lipoprotein cholesterol, TP = total protein.

## 4. Discussion

Our study investigated the relationship between the BMI/TP ratio and pregnancy outcomes in patients with GH (HDP). The analysis demonstrated a clear nonlinear, U-shaped association between the BMI/TP ratio and pregnancy survival, identified through restricted cubic spline modeling. Both low (<0.4) and high (>0.5) BMI/TP ratios were linked to an increased risk of adverse maternal outcomes, whereas moderate ratios (approximately 0.4–0.5) were associated with the most favorable pregnancy prognosis. This pattern indicates that either nutritional deficiency (reflected by low BMI/TP) or excessive metabolic burden (reflected by high BMI/TP) may contribute to vascular dysfunction and poor pregnancy outcomes. The threshold of 0.4 represented the inflection point detected in the spline model, while the cutoff value of 0.5, used for categorical grouping, was empirically derived from the cohort’s median BMI/TP ratio. Collectively, these findings highlight that the BMI/TP ratio exerts a threshold-dependent, nonlinear effect rather than a simple linear trend, emphasizing the importance of maintaining metabolic–nutritional balance during pregnancy.

During pregnancy, physiological hyperlipidemia occurs as part of maternal metabolic adaptation, supporting fetal growth and energy demands in later stages of gestation.^[12–14]^ However, when lipid metabolism becomes dysregulated, it contributes to adverse maternal and neonatal outcomes such as HDP, gestational diabetes, preterm birth, and fetal macrosomia.^[15,16]^ Importantly, studies have linked maternal lipid metabolism abnormalities with insulin resistance (IR) and inflammatory responses that exacerbate vascular dysfunction, a hallmark of HDP.^[17–20]^

Our findings extend this evidence by demonstrating that the BMI/TP ratio serves as an integrated index reflecting both maternal adiposity and protein nutritional status. The nonlinear, U-shaped association suggests that 2 distinct physiological extremes (insufficient protein reserves [low BMI/TP] and excessive adiposity with metabolic inflammation [high BMI/TP]) can both predispose to adverse outcomes. At low BMI/TP levels, inadequate protein and nutrient availability may impair vascular integrity and immune resilience. Conversely, elevated BMI/TP ratios likely indicate excessive metabolic load and pro-inflammatory activation, contributing to endothelial dysfunction and oxidative stress.

This balanced interpretation resolves the apparent discrepancy between protective and harmful effects by emphasizing that the relationship is threshold-dependent rather than monotonic. The observed pattern remained robust after multivariable adjustment, underscoring the independent prognostic value of the BMI/TP ratio in HDP.

Subgroup analysis further reinforced the utility of the BMI/TP ratio in risk prediction, revealing significant associations with comorbidities such as obesity, diabetes, cancer, arthritis, stroke, thyroid disorders, and heart failure (all *P* < .05). These findings indicate that the BMI/TP ratio not only mirrors adiposity and nutritional reserve but also integrates systemic inflammatory and metabolic disturbances that contribute to vascular injury in HDP.^[21–23]^ Thus, the BMI/TP ratio functions as a composite, nonlinear metabolic indicator encompassing both excess and deficiency components of maternal physiology, influenced by hepatic function, inflammation, and overall nutritional balance.^[11,24,25]^

Physiological changes in plasma volume during pregnancy can dilute serum protein concentrations, while estrogen-driven changes in hepatic lipoprotein metabolism contribute to elevated circulating TGs and very low-density lipoprotein levels.^[26–28]^ These changes are generally adaptive mechanisms supporting fetal growth; however, in HDP, this metabolic equilibrium may become disrupted, leading to endothelial injury, oxidative stress, and increased maternal and fetal risk.^[29–31]^ Within this context, an elevated BMI often reflects excess adipose tissue and metabolic overload, whereas reduced serum protein levels may indicate systemic inflammation, hepatic dysfunction, or nutritional insufficiency. Both extremes (metabolic excess and protein deficiency) can independently impair vascular function and compromise pregnancy outcomes.^[32,33]^

Specifically, low BMI/TP ratios likely reflect hypoalbuminemia and nutritional insufficiency, leading to reduced colloid osmotic pressure, increased interstitial edema, and impaired uteroplacental perfusion. Conversely, high BMI/TP ratios suggest excessive adiposity with concomitant low-grade inflammation and IR, which promote endothelial dysfunction, oxidative stress, and abnormal placental angiogenesis. These dual mechanisms explain why both insufficient protein reserves and excessive metabolic load may converge on similar pathophysiologic pathways that worsen HDP progression.

Furthermore, in conditions of IR, reduced lipoprotein lipase activity and elevated hepatic TG lipase expression can lead to increased circulating TGs and reduced HDL-C, contributing to the atherogenic lipid profile commonly observed in HDP.^[34–36]^ This constellation of lipid and protein imbalances provides a mechanistic explanation for the U-shaped relationship identified in our analysis: both inadequate protein synthesis and excessive lipid accumulation disrupt maternal vascular homeostasis, increase oxidative stress, and promote endothelial dysfunction. Therefore, the BMI/TP ratio effectively captures this bidirectional risk by integrating signals of metabolic load and nutritional reserve, reinforcing its role as a nonlinear, threshold-dependent biomarker of pregnancy outcomes.^[20,37–39]^

Collectively, these findings indicate that maintaining a balanced BMI/TP ratio (neither excessively low nor high) may represent an optimal metabolic state for favorable pregnancy outcomes. Routine measurement of BMI and total serum protein during late pregnancy is both feasible and cost-effective in clinical practice. Dynamic monitoring of this ratio could allow clinicians to identify women at elevated risk and implement timely interventions aimed at improving maternal and neonatal prognosis.

The strengths of this study include a relatively large cohort size, comprehensive subgroup analyses, and the integration of nonlinear modeling to elucidate threshold-dependent effects. Although no prospective sample size calculation was conducted, a post hoc power analysis based on the observed HR (0.30, 95% CI 0.16–0.57) demonstrated statistical power exceeding 0.90, confirming that the sample size (n = 306) was sufficient to detect clinically meaningful associations. However, as a single-center, retrospective study, this work is inherently subject to potential selection and information bias. Because only patients admitted to our institution were included, the cohort may not fully represent the broader population of women with GH. Residual confounding factors such as dietary patterns, physical activity, and genetic predisposition could not be fully controlled.

Moreover, dietary intake and lifestyle behaviors (which could influence both body composition and serum protein levels) were not systematically collected, limiting our ability to adjust for these variables in multivariable analyses. Additionally, a small proportion of laboratory data were missing; these were handled using case-wise exclusion and mean substitution procedures. Sensitivity analyses confirmed that these approaches did not materially influence the main results.

Another limitation involves the absence of external validation using an independent dataset. Although internal consistency across subgroup and nonlinear analyses was strong, external validation in diverse populations is needed to confirm the reproducibility and generalizability of these findings. Furthermore, certain subgroups (particularly those involving thyroid disorders and malignancy) contained limited event counts, which may have introduced statistical imprecision and resulted in wider CIs. The HR estimates derived from these smaller categories should therefore be interpreted with caution. Future validation in larger, multicenter cohorts is necessary to substantiate these subgroup-specific findings and enhance external validity.

It should also be noted that while multiple tables and figures were included to visualize subgroup and nonlinear associations, they were designed to be complementary rather than redundant. The principal conclusions were derived from consistent and statistically robust associations observed in adequately powered analyses, whereas results from smaller or less stable models were reported descriptively and interpreted conservatively. This approach enhances transparency and mitigates the risk of overstating spurious associations.

In summary, the BMI/TP ratio represents a nonlinear, threshold-dependent biomarker that integrates metabolic load and nutritional reserve. Prospective, multi-center studies with standardized data on nutrition and lifestyle, together with external validation cohorts, are warranted to confirm these findings and to explore whether targeted modulation of the BMI/TP ratio could improve maternal and fetal outcomes.

## 5. Conclusion

This study demonstrates that the BMI/TP ratio exhibits a nonlinear, U-shaped association with pregnancy survival in women with GH. Both low (<0.4) and high (>0.5) BMI/TP ratios were associated with increased risks of adverse maternal and fetal outcomes, while moderate ratios (approximately 0.4–0.5) were linked to the most favorable prognosis.

These findings suggest that maintaining an optimal metabolic–nutritional balance, as reflected by the BMI/TP ratio, may be critical for maternal and fetal health.

Given its simplicity, cost-effectiveness, and feasibility in routine clinical settings, the BMI/TP ratio may serve as a valuable prognostic indicator for individualized risk assessment and early intervention in hypertensive pregnancies.

Further prospective, multicenter studies are warranted to validate this nonlinear relationship and explore potential mechanisms underlying this association.

## Author contributions

**Conceptualization:** Dajun Cai.

**Data curation:** Dajun Cai, Shanshan Gao.

**Formal analysis:** Dajun Cai.

**Funding acquisition:** Dajun Cai.

**Investigation:** Dajun Cai, Jiahao Tang.

**Methodology:** Dajun Cai, Jiahao Tang.

**Software:** Qinqin Li.

**Validation:** Jiahao Tang, Shanshan Gao, Qinqin Li.

**Writing – original draft:** Dajun Cai, Xiaogai Qiao.

**Writing – review & editing:** Dajun Cai, Xiaogai Qiao.
